# Development and evaluation of food environment audit instrument: AUDITNOVA

**DOI:** 10.11606/s1518-8787.2019053001316

**Published:** 2019-10-10

**Authors:** Camila Aparecida Borges, Patricia Constante Jaime

**Affiliations:** I Universidade de São Paulo. Faculdade de Saúde Pública. Departamento de Nutrição. São Paulo, SP, Brasil

## Abstract

**OBJECTIVE:**

To develop and assess the reliability of an instrument that enables auditing information on consumer food environment indicators, such as availability, price, promotional and advertising strategies, and quantity of brands available, using the food recommendations adopted by the Dietary Guidelines for the Brazilian Population as a theoretical basis.

**METHODS:**

This is a methodological study in two phases: 1. development of the audit instrument and 2. assessment of its reliability and reproducibility *.* The Content Validity Index was estimated for each instrument item (>0.80 satisfactory). Inter-rater and test-retest reliability were assessed by percentage agreement and Kappa coefficients. Pearson’s correlation coefficient and Scatter-plots were used to measure the degree of linear correlation between two quantitative variables.

**RESULTS:**

The Content Validity Index was 0.91. Inter-rater and test-retest reliability were mostly high (Kappa> 0.80), for food availability indicators. Among the items that measure advertising, Kappa values for inter-rater reliability ranged from 0.57 to 1.00 and for the test-retest ranged from 0.18 to 0.90. Prices and quantity of brands showed a positive linear correlation between measurements performed by researcher 1 and 2 and between visits 1 and 2.

**CONCLUSIONS:**

AUDITNOVA is reliable for measuring aspects such as availability, price, quantity of brands, and advertising of foods available in the consumer food environment.

## INTRODUCTION

The food environment, in its multiple dimensions^1^ , influences food consumption and formation of eating habits^2 , 3^ . Strong evidence relates it to the chronic noncommunicable disease (CNCD) epidemic, especially obesity, in developed^4 , 5^ and developing countries^6^ . The food environment is also connected to increased body mass index^3^ and to two important dimensions that ensure food and nutritional security, healthy food access, and availability^7^ . Using a socioecological behavior approach, Glanz et al.^1^ suggest a conceptual model that divides the food environment into four main domains: community food environment, organizational food environment, information food environment, and consumer food environment. The latter refers to what consumers find inside and around retail food establishments, for example, healthy food availability, variety, price, promotions, shelf position, nutritional information, and advertising, determining factors in decision-making processes of food acquisition and consumption by the population^8 , 9^ . For example, a study in the United States found only 7% of the total food information in the weekly circulated leaflet from a supermarket chain was about fruit, 10% about vegetables, 10% about milk and dairy products, and 18% about cereals and grains; moreover, it showed information in these leaflets often influence consumers when making their food purchases^10^ .

The Dietary Guidelines for the Brazilian Population (DGBP) also recognizes the role of the food environment in promoting healthy eating and indicates six obstacles that hamper the adherence to current nutritional recommendations: information, advertising, time, cooking skills, cost, and food availability. Four of them are directly related to the food environment (information, advertising, cost, and availability), according to the model by Glanz et al.^1^ . Food environment aspects contributing to the health and quality of life of populations have been widely discussed in many countries and motivated the creation of indicator monitoring networks, such as the International Network for Food and Obesity/CNCD Research, Monitoring and Action Support (INFORMAS), a global network of public-interest organizations and researchers from 30 countries that aims to monitor, benchmark and support public and private sector actions to increase healthy food environments and reduce obesity, CNCD and health-related inequalities^11^ . INFORMAS proposes food environment monitoring indicators such as food nutritional composition, labeling, food advertising, supply chain, quantity and types of retail food establishments, prices, and investments in the food production chain^11^ .

As food environment contributes to obesity and CNCD, as shown in Brazilian technical documents^7 , 12 , 13^ and international networks^11^ , valid and reliable measures are necessary to study food environment^14^ . In the Brazilian scenario, research on this topic is recent^6 , 15 , 16^ , and studies need to expand production and collection of indicators, especially on the consumer’s food environment, which influences the behavior of food purchase and consumption^1^ .

None of the audit instruments already validated for the Brazilian context^17 , 18^ assess food availability and advertising according to the DGBP recommendations. Martins et al.^15^ , validating the Brazilian food environment audit instrument based on the adaptation of the study by Glanz et al.^14^ , approximate the food groups recommended by the DGBP, but explore only three indicators (availability, price, and quality) and disregard the latest version of the NOVA food classification, which divides them into four groups: *in natura* /minimally processed, culinary ingredients, processed foods, and ultra-processed foods^17^ . Brazilian instruments lack indicators that dialogue with national recommendations. Regarding this, identifying the density of commercial establishments that primarily sell ultra-processed foods, the food advertisements according to food type in line with NOVA, the sales of ultra-processed foods in places such as checkout aisles or the sales of *in natura* products at the establishment entrance and the promotional prices of healthy and unhealthy foods may contribute to the understanding of the relation between food consumption and environment in the face of the new Brazilian nutritional and epidemiological scenario^12^ .

The low reliability of the methods and instruments that propose to analyze the food environment and the current lack of objective criteria in the generation of indicators are barriers to understanding the mechanism of association between food environment, obesity, and food consumption^20^ . Thus, this study aims to develop and evaluate the reliability of a food environment audit instrument that enables capturing information of consumer food environment indicators such as availability, price, promotional and advertising strategies, and quantity of brands available, using the dietary recommendations adopted by the DGBP as theoretical basis.

## METHODS

This is a methodological study in two phases: 1. development of the audit instrument and 2. assessment of its reliability and reproducibility *.* The Ethics Committee of Faculdade de Saúde Pública of Universidade de São Paulo approved this study under CAAE number 69045917.5.0000.5421. All commercial establishments were aware of the informed consent form and voluntarily participated in the study.

### Audit Instrument Development

We designed the NOVA-based food environment audit instrument named AUDITNOVA in stages that involved systematic meetings of the research group; detailed analysis of Brazilian and international audit instruments and detailed analysis of the NOVA food classification proposed by Monteiro et al.^17^ . The processing of food identified by NOVA involves physical, biological and chemical processes applied after food is separated from nature and before it is eaten or prepared as dishes and meals. NOVA classifies all foods into four major groups:

- group 1: *in natura* or minimally processed foods. *In natura* foods are defined as edible parts of plants or animals after leaving nature and minimally processed foods are *in natura* foods altered by processes that include removal of inedible or unwanted parts and drying, crushing, grinding, fractionating, filtering, roasting, boiling, non-alcoholic fermentation, pasteurization, refrigeration, freezing, and vacuum packaging;
- group 2: culinary ingredients. Substances derived from group 1 or from nature by processes that include pressing, refining, grinding, and drying to make durable products used for cooking and preparing food at home or in restaurants;
- group 3: processed foods. They are essentially made by adding salt, oil, sugar, or other substances from group 2 to group 1;
- group 4: ultra-processed foods. Formulations made mainly or entirely from food-derived substances and additives, with little or no food from group 1^17^ .

We selected 66 foods for the AUDITNOVA with the highest frequency of acquisition by the Brazilian population according to data from the 2008-2009 Family Budget Survey (FBS)^21^ . The first version of the instrument had two blocks, one on food availability, prices, variety, and quality and another on advertising.

The first version of the instrument underwent the content validation process by a panel of judges with the participation of nine experts distributed among the following areas: food research, food advertising, and consumer protection. DGBP was the theoretical framework adopted during the panel of judges, especially Chapter 2 (“Choosing Food”) and Chapter 5 (“Understanding and Overcoming Obstacles”). The judges reviewed each AUDITNOVA item and assigned scores based on the 4-point Likert scale (1: disagree, 2: major revisions are necessary, 3: minor revisions are necessary, and 4: agree) for the clarity, relevance, pertinence, and representativeness attributes. In addition, the judges had a field for writing suggestions if the score was 3 or less. We estimated the content validity index (clear, relevant, pertinent, and representative) for each item and for each instrument block and considered it satisfactory when it reached 0.80 agreement among the judges. We discarded items below this percentage in the final version of the instrument^22^ .

The final version of AUDITNOVA, after analysis by the panel of judges, contains 14 blocks of questions divided into: block 1 — general information (municipality identification; evaluator identification; commercial establishment identification (sequential number assigned by the researcher); business name and address, date, collection start time and end time); block 2 — establishment type and products sold (type of commercial establishment, food groups sold according to NOVA, food groups sold primarily according to NOVA); block 3 — establishment entrance (fruit and vegetable section at the entrance of the store, food and advertisement availability in checkout aisles); block 4 — fruit and vegetable section (availability, unit of measure, current price in reais/kilo, and price type — normal or promotional); block 5 — meat, chicken, and fish section (availability, most expensive price in reais/kilo, cheapest price in reais/kilo, and price type); block 6 — dairy section (availability, quantity of brands available, most expensive price, cheapest price, and price type); block 7 — grocery section (availability, quantity of brands available, most expensive price, cheapest price, and price type); block 8 — canned food section (availability, quantity of brands available, most expensive price, cheapest price, and type of price); block 9 — bakery section (availability, quantity of brands available, most expensive price, cheapest price, and type of price); block 10 — frozen food section (availability, quantity of brands available, most expensive price, cheapest price, and price type); block 11 — beverage section (availability, quantity of brands available, most expensive price, cheapest price, and price type); block 12 — chocolate and snack section (availability, quantity of brands available, most expensive price, cheapest price, and price type); and finally, blocks 13 and 14 — advertisements inside and outside the establishment, respectively (Supplementary Archive).

### Reproducibility and Reliability of the Audit Instrument

We performed the AUDITNOVA reliability assessment study on a convenience sample in the metropolitan region of the city of São Paulo (SP), easily accessible by public transportation from Faculdade de Saúde Pública. We designed the neighborhood selection to maximize the ability to contrast supermarkets in neighborhoods with different income levels. To guarantee socioeconomic differences between them, we chose sites with different human development indexes (HDI): Pinheiros (HDI = 0.98), Higienópolis (HDI = 0.93), Belém (HDI = 0.91), and Sacomã (HDI) = 0.84). In each neighborhood, we selected 20 commercial establishments, among supermarkets, hypermarkets, and markets, totaling a sample of 80 establishments. Only seven refused to participate in the survey. Supermarkets, hypermarkets and markets have various food products available to consumers^23 , 24^ and are important equipment for measuring the reliability and reproducibility of a food environment audit instrument, as they enable the researcher to apply the instrument integrally.

To assess inter-rater reliability, two trained researchers independently visited the 73 commercial establishments in the region chosen for the study. Inter-rater reliability is used to assess the consistency of a measurement by different evaluators. To assess test-retest reliability, the same researchers revisited 41 sites 32 days after the initial observations. Test-retest reliability is used to assess the consistency of a measurement between two distinct moments.

For categorical variables, percentage agreement and Kappa coefficients assessed inter-rater and test-retest reliability. We quantified Kappa values using the following scale: 0.01 to 0.20, slight agreement; 0.21 to 0.40, fair agreement; 0.41 to 0.60, moderate agreement; 0.61 to 0.80, substantial agreement; 0.81 to 0.99, high agreement^25^ . We interpreted negative agreement values as a sign that evaluators agreed less on one item than expected due to chance — for example, a systematic disagreement among observers because diverse food items were available in the supermarkets. To assess the reliability of food availability according to NOVA, we grouped the 66 food items in the instrument into: group 1 — sum of all *in natura* /minimally processed foods; group 2 — sum of all culinary ingredients; group 3 — sum of all processed foods, and group 4 — sum of all ultra-processed foods.

For quantitative variables such as price and quantity of brands available, we first performed an exploratory analysis using scatter plots illustrating the linear fit and the quadratic fit. Scatter plots allow identifying patterns in data distribution and possible systematic and random errors depending on how dots are distributed along the line^26^ . Subsequently, we estimated Pearson’s correlation coefficient (r), which measures the degree of linear correlation between two quantitative variables. It is a dimensionless index with values between -1.0 and 1.0, which reflects the intensity of a linear relationship between two data sets. We estimated Pearson’s correlation coefficient between the pairs of variables collected by researcher 1 and researcher 2 and between the variables collected on the first and second visits. We estimated price and brand averages for each of the four food groups analyzed. We performed statistics on the statistical package Stata 14.

## RESULTS

At the content validation stage conducted by nine judges, the experts provided the necessary information to review the audit instrument and improve its content. The content validity index (CVI), which represents the average score for the clarity, relevance, pertinence, and representativeness attributes, was 0.91 for the entire instrument. Although the CVI was greater than 0.80 for most items, the suggestions provided by the experts were incorporated into the instrument because they are totally suitable according to the researcher’s assessment ( Table 1 ).

**Table 1:** AUDITNOVA content validation results: changes and reasons for the changes.

Item	Average CVI*	Change	Reasons for the change
Block 1 — Food availability, price, variety, and quality			

Item 1. Which *in natura* /minimally processed foods does the market sell?	0.97	Inclusion of new *in natura* foods and division of food items according to supermarket sections	To audit *in natura* /minimally processed foods often consumed by the Brazilian population and to facilitate data collection
Item 2. Is FV section located near the store entrance?	0.97	None	-
Item 3. *In natura* /minimally processed foods: availability and price	0.89	Inclusion of options for price type (normal or promotional) and unit of measure (kg, dozen, unit) for FV; removal of food quality item	Units of measure variation hindering price collection; low ability to distinguish food quality
Item 4. Which culinary ingredients does the market sell?	0.89	Inclusion of new items; distribution of items by supermarket section	To audit culinary ingredients frequently consumed by the Brazilian population and to facilitate data collection
Item 5 Culinary Ingredients: Availability and Price	0.97	Inclusion of price type (normal or promotional), quantity of brands, most expensive and cheapest price; standardization of items	To audit more details of culinary ingredients
Item 6 Which processed foods does the market sell?	0.97	Inclusion of new items; distribution of items by supermarket section	To audit processed foods often consumed by the Brazilian population and to facilitate data collection
Item 7. Processed foods: availability and price	0.92	Inclusion of price type (normal or promotional), quantity of brands, most expensive and cheapest price; standardization of items	To audit more details of processed foods
Item 8. Which ultra-processed foods does the market sell?	0.94	Inclusion of new items; distribution of items by supermarket section	To audit ultra-processed foods often consumed by the Brazilian population and to facilitate data collection
Item 9. Ultra-processed foods: availability and price	0.90	Inclusion of price type (normal or promotional), quantity of brands, most expensive and cheapest price; standardization of items	To audit more details of ultra-processed foods

Block 2 – publicity and advertising			

Item 1. Visual advertising encouraging FV purchase	0.88	Deleted and made into new items	New embedded items were able to capture the variability of advertisements
Item 2. Advertising type verified in FV section	0.89	Each type became a new questionnaire item	New embedded items were able to capture the variability of advertisements
Item 3. Appeal and reason for the advertising in the FV section	0.97	Each appeal and reason became a new item in the questionnaire.	New embedded items were able to capture the variability of advertisements
Item 4. Visual advertising that encourages the purchase of sugary drinks such as added sugar juices, nectars, and soft drinks.	0.94	Deleted and made into new items to evaluate ultra-processed advertising in general	New embedded items were able to capture the variability of advertisements
Item 5. Types of sugary drink advertisements	0.92	Deleted and made into new items to evaluate ultra-processed advertising in general	New embedded items were able to capture the variability of advertisements
Item 6. Appeal and reason for the advertising in the sugary drink section	0.89	The most relevant appeals and reasons became new items in the questionnaire.	New embedded items were able to capture the variability of advertisements
Item 7. Visual advertising encouraging the purchase of crackers, cookies and cornmeal snacks in the sections where these foods can be found.	0.92	Deleted and made into new items to evaluate ultra-processed advertising in general	New embedded items were able to capture the variability of advertisements
Item 8. Crackers/Cookies/Cornmeal snacks: Types of Advertisements	0.86	Deleted and made into new items to evaluate ultra-processed advertising in general	New embedded items were able to capture the variability of advertisements
Item 9. Appeal and reason for the advertising in the cookie/cracker/snack section	0.86	The most relevant appeals and reasons became new items in the questionnaire.	New embedded items were able to capture the variability of advertisements
Item 10. Are there any health promotion or advertising messages related to food and health in the establishment?	0.83	No changes, but applied to *in natura* /minimally processed and ultra-processed foods	This theme applies to many types of healthy and unhealthy foods
Item 11. Write down the messages	0.81	Deleted	New embedded items were able to capture the variability of advertisements
Item 12. Do the checkout aisles have magazines/posters/folders with news about fad diets, superfoods, diet versus light and/or other food and health news?	0.97	None	-
Item 13. Do the checkout aisles sell chocolates, treats, soft drinks, energy drinks or other types of ultra-processed products?	0.92	Each food was detailed in a new item.	More detailed characterization of the *checkout* aisle
Item 14. Do the checkout aisles have ultra-processed food advertising?	0.94	None	-
Total CVI of the instrument	0.91	-	-

FV: fruits and vegetables; *checkout* : areas where the cash registers of the commercial establishments are located

*The CVI (content validity index) represents the average score given by the nine expert judges for the clarity, relevance, pertinence, and representativeness attributes.

During the audit process, both trained researchers’ first visits to the 73 establishments occurred in an average of 41 days (standard deviation = 11.8 days). The average application time of AUDITNOVA was 90 minutes (standard deviation = 7.0 minutes). The researchers’ second visit occurred between 32 and 47 days after the first collection, with an average of 39.5 days (standard deviation = 4.8 days).

Table 2 shows the inter-rater and test-retest reliability results for the 66 audited foods and for the four NOVA food groups. Most foods had Kappa values higher than 0.80 (substantial agreement) for both inter-rater and test-retest. Analyzing the NOVA groups, we found *in natura* /minimally processed foods showed moderate Kappa values (0.41–0.60) for both inter-rater and test-retest. The other three food groups (culinary ingredients, processed foods, and ultra-processed foods) had Kappa values above 0.70 for inter-rater reliability, ranging from 0.57 to 0.64 for test-retest reliability, which indicates moderate agreement between visits.

**Table 2:** Reliability of the AUDITNOVA instrument according to indicators of food availability.

Types of Foods Available	Inter-rater reliability			Test-retest reliability		
	
	n	agreement %	Kappa	n	agreement %	Kappa
*In natura* /Minimally Processed Foods	72	59.7	0.55	41	55.8	0.52
Orange	73	100.0	1.00	41	92.7	0.88
Banana	73	94.5	0.72	41	82.9	0.72
Formosa papaya	72	100.0	1.00	41	78.5	0.61
Fuji apple	73	93.1	0.70	41	90.2	0.79
Watermelon	72	97.2	0.93	41	85.4	0.74
Other fruits	73	98.6	0.85	41	97.6	0.94
Tomato	73	93.1	0.83	41	90.2	0.84
Onion	73	98.6	0.85	41	92.7	0.84
Lettuce	73	100.0	1.00	41	87.8	0.78
Carrot	73	98.6	0.90	41	78.0	0.64
Zucchini	73	91.8	0.75	41	87.8	0.79
Chayote	73	98.6	0.91	41	92.7	0.86
Mix of parsley, spring onion, and cilantro	73	95.9	0.88	41	82.9	0.71
Other vegetables	73	97.3	0.78	41	92.7	0.86
Potato	73	98.6	0.90	41	85.4	0.72
Cassava	73	98.6	0.97	41	65.8	0.44
Other roots and tubers	73	94.5	0.64	41	95.1	0.90
Sweet corn	73	94.5	0.87	41	73.2	0.57
Eggs	73	94.5	0.84	41	75.6	0.6
Other eggs	73	97.3	0.90	41	85.4	0.74
Prime beef	73	100.0	1.00	41	87.8	0.81
Choice beef	73	98.6	0.97	41	87.8	0.81
Chicken	73	98.6	0.96	41	80.5	0.68
Chicken Breast	73	98.6	0.85	41	95.1	0.90
Fish	73	98.6	0.93	41	100.0	1.00
Cow milk	73	100.0	1.00	41	97.6	0.95
Pinto beans	73	100.0	1.00	41	95.1	0.91
Black turtle beans	73	100.0	1.00	41	90.2	0.82
White rice	73	100.0	1.00	41	97.6	0.95
Wheat flour	73	100.0	1.00	41	100.0	1.00
Cassava flour	73	98.6	0.79	41	87.8	0.73
Spaghetti	73	98.6	0.93	41	100.0	1.00
Raw peanut	73	90.4	0.70	41	75.6	0.56
500 ml Water	73	100.0	^a^	41	97.6	0.95
5-liter gallon Water	73	100.0	1.00	41	73.2	0.55
Culinary Ingredients	73	93.1	0.87	41	70.7	0.64
Butter	73	100.0	1.00	41	92.7	0.86
Soybean oil	73	100.0	^a^	41	95.1	0.89
Olive oil	73	100.0	1.00	41	97.6	0.93
Refined salt	73	98.6	0.79	41	100.0	1.00
Sanding Sugar	73	94.5	0.88	41	80.5	0.68
White Sugar	73	100.0	^a^	41	100.0	1.00
Processed foods	73	94.5	0.89	41	68.3	0.61
Bacon	73	98.6	0.91	41	92.7	0.86
Dried meat	73	97.3	0.93	41	80.5	0.70
Cheese	73	100.0	^a^	41	97.6	0.94
Canned Corn	73	100.0	1.00	41	97.6	0.95
Tomato purée	73	100.0	1.00	41	100.0	1.00
Canned Sardines	73	98.6	0.79	41	97.6	0.95
Bread roll	73	100.0	1.00	41	82.9	0.68
Ultra-Processed Foods	73	82.2	0.76	41	61.0	0.57
Hot dog sausage	73	100.0	1.00	41	95.1	0.91
Pork Sausage	73	93.1	0.80	41	90.2	0.82
Fermented milk	73	98.6	0.95	41	92.7	0.86
Instant noodle	73	98.6	0.88	41	90.2	0.80
Ready-made seasoning	73	100.0	1.00	41	100.0	1.00
Sliced bread	73	97.3	0.78	41	90.2	0.79
Breakfast Cereals	73	98.6	0.88	41	97.6	0.94
Frozen pizza	73	100.0	1.00	41	95.1	0.90
Ice cream	73	98.6	0.94	41	85.4	0.73
Regular Can Soda^a^	73	100.0	1.00	41	100.0	1.00
Regular 2-liter Soda^b^	73	100.0	1.00	41	100.0	1.00
Zero/zero sugar/diet soda	73	100.0	1.00	41	97.6	0.94
Nectar	73	100.0	1.00	41	100.0	1.00
Fruit juice drink	73	100.0	1.00	41	100.0	1.00
Cornmeal snacks	73	100.0	1.00	41	100.0	1.00
Sandwich cookie	73	100.0	^a^	41	100.0	^a^
Chocolate	73	95.9	0.80	41	92.7	0.85
Candies	73	97.3	0.94	41	97.5	0.96

^a^ Statistics could not be estimated because the cross tabulation had two or fewer levels.

^b^ Soda with added sugar.

Table 3 shows the inter-rater and test-retest reliability results for the sold and primarily sold foods according to NOVA indicators, fruit and vegetable section at the entrance of the establishment and price type (normal or promotional) for foods grouped according to NOVA. Despite the high agreements observed for all indicators, Kappa values in both inter-rater and test-retest reliability were reasonable (0.21–0.40) and moderate (0.41–0.60), showing the low capacity of agreement of some, such as priority sale of processed foods and price type of ultra-processed foods.

**Table 3:** Reliability of the AUDITNOVA instrument according to food availability indicators, and price types according to NOVA.

Indicators of availability of food groups and price type according to NOVA	Inter-rater reliability			Test-retest reliability		
	
	n	agreement %	Kappa	n	agreement %	Kappa
FV at the store entrance	73	95.9	0.92	41	97.6	0.95
Food groups sold						
*In natura* /minimally processed Foods	73	100.0	*	41	100.0	*
Culinary ingredients	73	100.0	*	41	100.0	*
Processed foods	73	100.0	*	41	100.0	*
Ultra-processed Foods	73	100.0	*	41	100.0	*
Primarily sold food groups						
*In natura* /minimally processed foods	73	75.3	0.69	41	90.2	0.78
Culinary ingredients	73	97.3	0.75	41	95.1	0.88
Processed foods	73	87.7	0.36	41	95.1	0.90
Ultra-processed Foods	73	91.8	0.78	41	100.0	1.00
Price type (normal or promotional)						
*In natura* /minimally processed Foods	73	93.0	0.90	41	93.6	0.89
Culinary ingredients	73	81.1	0.77	41	82.1	0.78
Processed foods	73	71.4	0.61	41	70.4	0.58
Ultra-Processed Foods	73	61.7	0.56	41	52.6	0.48

FV: Fruits and Vegetables

*Statistics could not be estimated because the cross tabulation had two or fewer levels.

Table 4 shows the inter-rater and test-retest reliability results for the advertising variables in AUDITNOVA. The inter-rater reliability obtained a higher number of Kappa coefficients above 0.80 than the test-retest. For inter-rater reliability, Kappa values ranged from 0.57 to 1.00, and for the test-retest, from 0.18 to 0.90 — the highest disagreement.

**Table 4:** Reliability of the AUDITNOVA instrument according to advertising indicators, and food advertising strategies according to the NOVA classification.

Types of Advertisements and Advertising Strategies	Inter-rater reliability			Test-retest reliability		
	
	n	agreement %	Kappa	n	agreement %	Kappa
*Checkout aisle advertisements*						
In *natura* /minimally processed food advertisements of different types	73	89.0	0.57	41	90.2	0.77
UPF food advertising of different types	73	93.1	0.81	41	92.7	0.83
Tabloid	73	94.5	0.90	41	94.5	0.90
Folder or leaflet	73	98.6	0.88	41	95.1	0.79
Approaches in physical space						
*In natura* food flags	73	87.7	0.65	41	87.8	0.72
*In natura* food banner/posters	72	84.7	0.60	41	82.5	0.52
*In natura* food displays	73	89.0	0.65	41	87.8	0.46
*In natura* food tabloids	73	87.7	0.75	41	65.8	0.39
*In natura* food recipes folder/leaflet	73	91.8	0.65	41	90.2	0.55
UPF food displays	73	100.0	1.00	41	95.2	*
UPF food promotional islands	73	86.3	0.67	41	68.3	0.37
UPF food endcaps	73	82.2	0.66	41	72.5	0.48
UPF food tasting counters	73	97.3	0.74	41	95.1	*
Message types and consumer appeals						
Food functional property with *in natura* food	73	98.6	0.95	41	87.8	0.72
Physical activity and *in natura* food	73	98.6	0.66	41	95.1	0.62
Wellness, good mood, self-esteem with *in natura* /minimally processed food	73	91.8	0.62	41	92.7	0.70
Health claim with *in natura* food	72	95.8	0.84	41	82.9	0.72
Appeal to practicality with *in natura* /minimally processed food	73	94.5	0.68	41	92.7	0.67
Highlight in *in natura* /minimally processed food flavor, odor, color, or texture	72	94.4	0.84	41	87.8	0.48
Health and wellness claim with UPF food	73	86.3	0.72	41	78.0	0.58
Appeal for practicality with UPF food	73	84.9	0.62	41	56.1	0.23
Functional property with UPF	73	84.9	0.68	41	72.5	0.41
Highlight in UPF food flavor, odor, color, or texture	73	90.4	0.80	41	65.8	0.43
Advertisements about tastings, giveaways, promotions, and releases						
Take 3, pay 2 with *in natura* food	73	98.6	*	41	97.6	*
*In natura* /minimally processed product launches	72	98.6	*	41	*	*
Natura/minimally processed *giveways* or tie-in sales	73	95.9	0.64	41	*	*
UPF free samples	73	98.6	*	41	95.6	*
Take 3, pay 2 with UPF	73	87.7	0.71	41	78.0	0.46
UPF releases	73	78.1	0.62	41	53.6	0.18
UPF giveaways or tie-in sales	73	79.4	0.61	41	73.2	0.46
Advertising in general with culinary ingredients	73	86.3	0.70	41	61.0	0.18
Processed food advertising in general	73	80.8	0.65	41	61	0.20
Advertisements outside the establishment						
Advertising in general with *in natura* /minimally processed foods	73	90.4	0.70	41	78.0	0.61
Advertising in general of culinary ingredients	73	94.5	0.77	41	80.5	0.66
Advertising of processed foods in general	73	94.5	0.82	41	75.6	0.58
UPF advertising outside the establishment	73	93.1	0.85	41	78.5	0.60

*Checkout* : areas where cash registers are located in commercial establishments; UPS: Ultra-Processed Foods

*Statistics could not be estimated because the cross tabulation had two or fewer levels.

**Figure 1:**
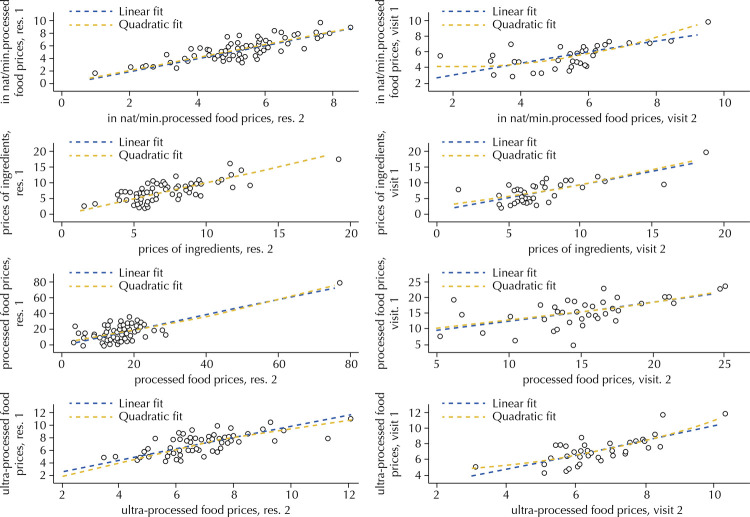
Relationship between food price variables according to NOVA collected by researchers 1 and 2 at the first and second visit.

Figure 1 shows the scatter plots illustrating the relationship between food price variables according to NOVA collected by researchers 1 and 2 and collected on the first and second visit. The inclinations of the lines show a positive correlation in all cases.

Res: researcher; in nat/min.processed: *in natura* and minimally processed

**Figure 2:**
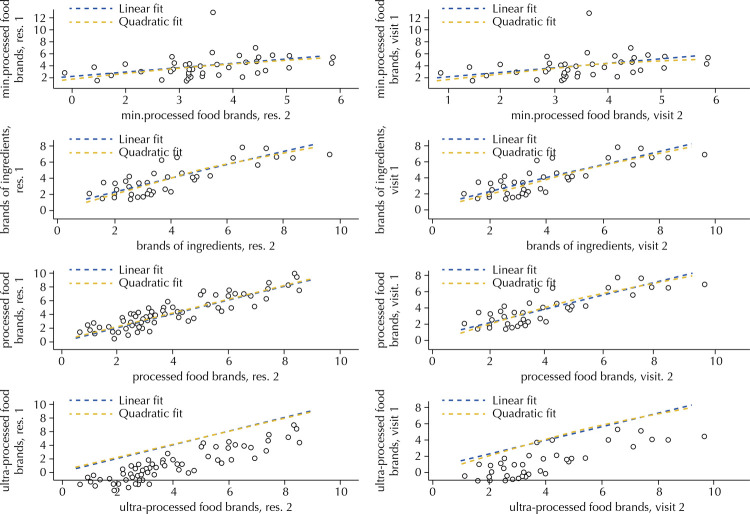
Relationship between food price variables according to NOVA collected by researchers 1 and 2 at the first and second visit.

Figure 2 shows the scatter plots illustrating the relation between the variables quantity of food brands according to NOVA, collected by researchers 1 and 2 and collected at the first and second visit. The inclinations of the lines show a positive correlation in all cases.

Res: researcher; min .processed: minimally processed

Table 5 shows the average values of price and quantity of brands found by researchers 1 and 2 at each visit, as well as the Pearson correlation (r) values of the pairs of variables analyzed. Although all correlation values were positive, the quantity of minimally processed food brands had the lowest r values both among evaluators and between visits.

**Table 5:** Mean and standard deviation (SD) of price and quantity of brands collected by researchers 1 and 2 and at visits 1 and 2, and Pearson’s correlation coefficient (r) values between pairs of variables.

Quantitative variables	Inter-rater			Test-retest		
	
	Mean (SD) Researcher 1	Mean (SD) Researcher 2	r	Mean (SD) Visit 1	Mean (SD) Visit 2	r
*In natura* /minimally processed food price	5.34 (1.51)	5.28 (1.42)	0.98	5.51 (1.26)	5.35 (1.47)	0.82
Price of ingredients	7.17 (2.68)	7.15 (2.61)	0.99	7.12 (2.66)	7.11 (2.86)	0.90
Processed food price	16.14 (8.41)	16.34 (8.59)	0.97	15.09 (4.23)	15.04 (4.28)	0.65
Ultra-processed food price	6.85 (1.53)	6.67 (1.59)	0.95	6.93 (1.60)	6.63 (1.36)	0.84
Minimally processed food brands	3.45 (1.19)	3.51 (1.62)	0.75	3.91 (1.73)	3.46 (1.10)	0.47
Ingredient Brands	3.88 (2.05)	3.85 (2.01)	0.98	3.91 (1.89)	4.01 (2.09)	0.93
Processed food brands	3.09 (1.18)	3.10 (1.23)	0.96	3.35 (1.15)	3.13 (1.20)	0.89
Ultra-processed food brands	4.19 (1.35)	4.00 (1.21)	0.97	4.32 (1.06)	4.24 (1.11)	0.92

## DISCUSSION

The food environment audit instrument developed in this study, AUDITNOVA, had high inter-rater and test-retest reliability, which ensures that it is a reliable instrument for studies aimed at working with food environment indicators based on the NOVA food classification proposed by Monteiro et al.^17^ . We carefully selected the indicator foods of the four groups proposed in NOVA because Brazilians frequently purchase them, according to national surveys, and DGBP recommends them. These foods included in AUDITNOVA may assess retail establishments regarding their availability of healthy and unhealthy foods according to Brazilian guidelines^12^ . In addition, information about price, quantity of brands and advertising will enable assessing the consumer’s food environment in detail, observing the barriers and conveniences that consumers face when choosing their food^1^ . Most of the indicators in this instrument are appropriate for the planning of policy programs aimed at modifying the environment, assessing intervention needs and population needs when faced with food availability, and serving as evaluation, surveillance and advocacy indicators for other actions based on the consumer’s food environment^20^ .

High inter-rater reliability shows the definitions and instructions in the measurement manual and training methods were sufficient to prepare observers to collect high-quality data. The high test-retest reliability in most of the indicators suggests only minor changes in food availability, price, quantity of brands, and advertising strategies occur over the data collection period. Thus, the measures collected with AUDITNOVA generated a stable estimate of the consumer’s food environment. However, the availability and price of *in natura* /minimally processed foods often change over the seasons; therefore, whenever the instrument is reapplied, repeated observations should be considered to assess or control seasonal effects^14^ .

In Brazil, studies on food environment are recent, Martins et al.^15^ and Duran et al.^16^ developed and validated pioneering instruments for auditing the community food environment and the consumer food environment. The audit instrument validated by Martins et al.^15^ is an adaptation of the one developed by Glanz et al.^14^ to measure the consumer food environment, specifically retail food establishments, and to assess aspects such as food availability, price, and quality with a food list guided by the food pyramid and the degree of processing. However, it disregards the full version of the NOVA classification. Duran et al.^16^ proposed an audit instrument designed to audit retail food establishments and restaurants and to measure aspects such as availability, variety, quality, price, and advertising of healthy food indicators such as fruits and vegetables and of unhealthy food indicators such as ultra-processed foods. The main differences of the AUDITNOVA developed and validated in this study comparing with the other two Brazilian instruments were the full use of the NOVA classification in the food item selection, the expansion of advertising and promotional strategies by food groups, the availability of 66 food items (including culinary ingredients and processed foods), the inclusion of strategic aspects of the consumer food environment (such as checkout aisles, endcaps, and islands), and the collection of information on normal or promotional prices, determining factors in the food acquisition by the population^1 , 9 , 14 , 20^ .

The main indicators proposed in this instrument showed substantial and high Kappa values. Kappa values were moderate for the indicator of availability of *in natura* /minimally processed foods, especially in the test-retest, but Kappa values were substantial when evaluating food items in isolation. However, the seasonality and the low variety of *in natura/* minimally processed foods in supermarkets and markets compared with street markets, big retail markets and farmer’s markets may have influenced the indicator reliability^24 , 27^ .

AUDITNOVA enables measuring the different food information sources available in the consumer food environment in detail, dividing the types of advertising according to the NOVA’s four food groups. The DGBP recognizes that the publicity and information available in the consumer food environment can become an obstacle for the population to reach the food recommendations^12^ , because large food industries, especially the ultra-processed food ones, use the advertisements to sell more products, not to educate consumers^28^ .

The World Health Organization also recognizes that the massive advertising campaigns adopted by the food industries, especially those aimed at children and with different appeals (health, fitness, convenience, releases, children’s characters, among others), affect these individuals’ health. Thus, countries should review the regulatory processes regarding the propagation of these advertisements on packaging and in the mainstream media^28^ . Concerning this, the development of audit instruments that provide an overview of these advertising practices in the consumer food environment and corroborate the DGBP will be essential for the advancement of public policies and regulation. Dietary environment indicators that enable producing more evidence about their influence are part of the strategy to face obesity and CNCD^5^ .

The advertising variables measured by AUDITNOVA showed higher inter-rater reliability than in the test-retest, including many values that could not be computed due to the low availability of advertisements in the establishments. Duran et al. also observed this fact in their study,^16^ , and it may indicate the researcher’s difficulty in identifying the different advertising strategies available in the retail establishment and in knowing how to distinguish, in particular, the types of appeals that these advertisements bring. Advertisements with Kappa values lower than 0.40 in the test-retest were: tabloids with *in natura* /minimally processed food advertisements, promotional islands with ultra-processed foods, appeal to the convenience of ultra-processed foods, ultra-processed food launches, and advertisements of culinary ingredients and processed foods, in general. One hypothesis to improve the reliability of this indicator would be to conduct more than one field researcher training throughout the audit process to reaffirm the different types of appeal and approaches of food advertising in retail and/or to expand the sample of audited establishments to increase the prevalence of these types of advertising. However, researchers can still use the instrument in the field. As the instrument is built in independent blocks, they will be free to select the indicators that best fit their research goals.

The variables price and quantity of brands showed positive correlations between the measurements made by researchers 1 and 2 and visits 1 and 2. Both price and quantity of brands influence consumers when buying food^29^ . Measuring these aspects reliably, even over a certain time range, is essential for the use of the instrument in the monitoring and mapping of these indicators in different commercial establishments and in different social realities.

Although this study disregarded the food environment throughout the year, at certain times (for example, Christmas, Easter, Father’s Day, and Mother’s Day), price, availability, and especially advertising indicators^a^ may vary beyond expected due to advertising campaigns and new products available on those dates. Therefore, the researcher must assess the necessity of applying the instrument in these periods.

Some of the strengths of this study are the content validation process prepared by a panel of judges specialized in food environment and food advertising, and the use of the NOVA food classification as a theoretical and analytical framework. In addition, the use of Brazilian databases, such as that of *Pesquisa de Orçamentos Familiares* (POF – Brazilian Family Budget Survey), provided subsidies for selecting foods that are frequently purchased by the Brazilian population. Another strength of the study is the presence of foods in greater variety in relation to Brazilian instruments, enabling the grouping according to NOVA, as well as the inclusion of more complete information on advertising, prices and quantity of brands, which may provide a more detailed overview of food environment for researchers who will use the instrument.

One of the limitations of this study is the convenience sample of only one Brazilian city and the low variety of audited business types (supermarkets, hypermarkets, and markets only). This sample does not represent the municipality and the country; however, neighborhoods have significant socioeconomic variations that may impact the food availability audited. Another limitation is the lack of evaluation of seasonal differences during the year. The instrument evaluated only retail establishments used by the population for food purchase and not for immediate consumption, such as bars and restaurants. As many individuals eat out in Brazil^21^ , developing and validating appropriate instruments to audit these places according to the new national food recommendations is necessary. This study did not use a quality indicator of retail food establishments based on possible scores generated by the instrument, a fact recognized as important, which will be considered for future studies.

The instrument developed, AUDITNOVA, proved to be reliable for audits in the food environment, especially in the consumer food environment, as it enables an overview of types of retail equipment in the territory and a broad analysis of the main determinants that contribute to supporting the population to choose healthier food. AUDITNOVA is reliable for measuring aspects such as availability, price, quantity of brands, and food advertising. Associations between food environment, food consumption, and obesity are becoming more frequent; however, reliable data collection instruments are needed to reach these results. The development and validation of a food environment audit instrument based on the recommendations presented in the DGBP dialogues with other Brazilian policies and supports the development of evidence that allows us to rethink the role of the food environment in availability, access, and consequently, food and nutritional security of the Brazilian population. We published the data collection training manual developed in this investigation and the AUDITNOVA instrument, which are available for download at: http://colecoes.sibi.usp.br/fsp/items/show/3364#?c=0&m=0&s =0&cv=0.

Research supported by the International Development Research Center (IDRC) and the Brazilian Institute of Consumer Protection (Instituto Brasileiro de Defesa do Consumidor – IDEC) for data collection and São Paulo Research Foundation (Process 2016/12766-6)

## Footnotes

a Criança e Consumo. Páscoa para quê? Responda prá gente saber! [cited 2019 Mar 27]. Available from: http://criancaeconsumo.org.br/noticias/pascoaparaque/
