# Supplementary material for: Development and evaluation of food environment audit instrument: AUDITNOVA
Source: Rev Saude Publica. 2019 Oct 10;53:91. doi: 10.11606/s1518-8787.2019053001316 (PMC9586434; doi:10.11606/s1518-8787.2019053001316)
Supplement: Translation

# Translation: Desenvolvimento e avaliação de instrumento de auditoria do ambiente alimentar: AUDITNOVA

**Authors:** Camila Aparecida Borges, Patricia Constante Jaime

**Affiliations:** I Universidade de São Paulo. Faculdade de Saúde Pública. Departamento de Nutrição. São Paulo, SP, Brasil

**Keywords:** Guias Alimentares, Produtos de Venda Direta ao Consumidor, Defesa do consumidor, Controle e Fiscalização de Alimentos e Bebidas, Disseminação de Informação, Segurança Alimentar e Nutricional, Estudos de Validação

## Abstract

**OBJETIVO:**

Desenvolver e avaliar a confiabilidade de um instrumento de auditoria do ambiente alimentar do consumidor que possibilite captar informações sobre indicadores do ambiente alimentar do consumidor como disponibilidade, preço, estratégias promocionais e publicitárias e quantidade de marcas disponíveis, utilizando como base teórica as recomendações alimentares adotadas pelo Guia Alimentar para a População Brasileira.

**MÉTODOS:**

Trata-se de um estudo metodológico em duas fases: 1. desenvolvimento do instrumento de auditoria e 2. avaliação de sua confiabilidade e reprodutibilidade *.* Foi calculado o índice de validade de conteúdo para cada item do instrumento (satisfatório > 0,80). A confiabilidade interavaliadores e teste-reteste foram avaliadas por percentual de concordância e coeficientes de Kappa. O coeficiente de correlação de Pearson e scatter-plots foram utilizados para medir o grau da correlação linear entre duas variáveis quantitativas.

**RESULTADOS:**

O índice de validade de conteúdo total foi de 0,91. A confiabilidade interavaliadores e teste-reteste foram altas (Kappa >0,80), em sua maioria, para indicadores de disponibilidade de alimentos. Entre os itens que medem a publicidade, a confiabilidade interavaliadores apresentou valores de Kappa de 0,57 a 1,00, enquanto o teste-reteste apresentou Kappas de 0,18 a 0,90. Preços e quantidade de marcas apresentaram correlação linear positiva entre as medidas realizadas pelo pesquisador 1 e 2 e entre as visitas 1 e 2.

**CONCLUSÕES:**

O AUDITNOVA é confiável para mensurar aspectos como disponibilidade, preço, quantidade de marcas e publicidade de alimentos disponíveis no ambiente alimentar do consumidor.

## INTRODUÇÃO

O ambiente alimentar, em suas múltiplas dimensões^1^ , influencia o consumo alimentar e a formação de hábitos alimentares^2 , 3^ . Têm surgido fortes evidências de sua relação com a epidemia de doenças crônicas não transmissíveis (DCNT), em especial, a obesidade, em países desenvolvidos^4 , 5^ e em desenvolvimento^6^ . Ele também está relacionado ao aumento do índice de massa corporal^3^ e a duas dimensões importantes para garantia da segurança alimentar e nutricional, o acesso e a disponibilidade de alimentos saudáveis^7^ . Utilizando uma abordagem sociológica de comportamento, Glanz et al.^1^ propõem um modelo conceitual que divide o ambiente alimentar em quatro domínios principais: ambiente alimentar comunitário, ambiente alimentar organizacional, ambiente alimentar da informação e ambiente alimentar do consumidor. Esse último se refere ao que os consumidores encontram dentro e ao redor dos comércios varejistas de alimentos, por exemplo, disponibilidade de alimentos saudáveis, variedade, preço, promoções, posição na prateleira, informações nutricionais e publicidade, itens determinantes nos processos decisórios de aquisição e consumo de alimentos pela população^8 , 9^ . Por exemplo, um estudo realizado nos Estados Unidos verificou que, do total de informações alimentares presentes no folheto circulado semanalmente em uma rede de supermercados, somente 7% eram sobre frutas, 10% sobre hortaliças, 10% sobre leite e derivados e 18% sobre cereais e grãos; além disso, mostrou que os consumidores eram frequentemente influenciados pelas informações veiculadas nestes folhetos no momento de fazer suas compras de alimentos^10^ .

O Guia Alimentar para a População Brasileira (GAPB) também reconhece o papel do ambiente alimentar na promoção da alimentação saudável e aponta seis obstáculos que dificultam a adesão às recomendações nutricionais vigentes: informação, publicidade, tempo, habilidades culinárias, custo e disponibilidade de alimentos. Quatro deles têm relação direta com o ambiente alimentar (informação, publicidade, custo e disponibilidade), segundo o modelo de Glanz et al.^1^ . Os aspectos do ambiente alimentar que contribuem para a saúde e qualidade de vida de populações têm sido amplamente discutidos por diversos países e motivado a criação de redes de monitoramento de indicadores, como no caso da Rede Internacional de Pesquisa em Alimentos e Obesidade/DCNT (Informas), uma rede global de organizações de interesse público e pesquisadores de 30 países que visa monitorar, avaliar e apoiar ações dos setores público e privado para fomentar ambientes alimentares saudáveis e reduzir a obesidade, as DCNT e as desigualdades relacionadas a essas condições de saúde. Entre os indicadores de monitoramento de ambiente alimentar propostos pelo Informas estão àqueles que dizem respeito à composição nutricional dos alimentos, rotulagem, publicidade de alimentos, rede de abastecimento, quantidade e tipos de comércios varejistas de alimentos, preços e investimentos na cadeia produtiva de alimentos^11^ .

Devido à contribuição do ambiente alimentar para a obesidade e as DCNT, como apontado por documentos técnicos nacionais^7 , 12 , 13^ e redes internacionais^11^ , é necessário estudá-lo por meio de medidas válidas e confiáveis^14^ . No cenário brasileiro, são recentes as pesquisas sobre esse tema^6 , 15 , 16^ , fazendo-se necessário ampliar a produção e coleta de indicadores, em especial sobre o ambiente alimentar do consumidor, o qual influencia o comportamento de compra e consumo de alimentos^1^ .

Há instrumentos de auditoria validados para o contexto brasileiro^17 , 18^ , mas nenhum deles avalia a disponibilidade de alimentos e a publicidade segundo as recomendações do GAPB. Martins et al.^15^ , ao validar um instrumento brasileiro de auditoria de ambiente alimentar baseado na adaptação do estudo de Glanz et al.(14), fazem uma aproximação dos grupos alimentares recomendados pelo GAPB, porém exploram somente três indicadores (disponibilidade, preço e qualidade) e não utilizam a versão mais atual da classificação de alimentos NOVA, que os divide em quatro grupos: *in natura* /minimamente processados, ingredientes culinários, alimentos processados e alimentos ultraprocessados^17^ . Verifica-se nos instrumentos brasileiros a falta de indicadores que dialoguem com as recomendações nacionais. Nesse sentido, identificar a densidade de estabelecimentos comerciais que vendem prioritariamente alimentos ultraprocessados, propagandas de alimentos segundo tipo de alimento de acordo com a NOVA, vendas de alimentos ultraprocessados em locais como caixas ou venda de produtos *in natura* na entrada do comércio e preços promocionais de alimentos saudáveis e não saudáveis poderá contribuir para o entendimento da relação entre consumo alimentar e ambiente diante do novo cenário nutricional e epidemiológico brasileiro^12^ .

A baixa confiabilidade dos métodos e instrumentos que se propõem a analisar o ambiente alimentar e a atual falta de critérios objetivos na geração dos indicadores é uma barreira para entender o mecanismo de associação entre ambiente alimentar, obesidade e consumo alimentar^20^ . Sendo assim, o objetivo do presente estudo é desenvolver e avaliar a confiabilidade de um instrumento de auditoria do ambiente alimentar que possibilite captar informações sobre indicadores do ambiente alimentar do consumidor como disponibilidade, preço, estratégias promocionais e publicitárias e quantidade de marcas disponíveis, utilizando como base teórica as recomendações alimentares adotadas pelo Guia Alimentar para a População Brasileira.

## MÉTODOS

Trata-se de um estudo metodológico em duas fases: 1. desenvolvimento do instrumento de auditoria e 2. avaliação de sua confiabilidade e reprodutibilidade *.* O estudo foi aprovado pelo Comitê de Ética da Faculdade de Saúde Pública da Universidade de São Paulo sob número do CAAE 69045917.5.0000.5421. Todos os estabelecimentos comerciais foram informados do termo de consentimento livre e esclarecido e participaram voluntariamente do estudo.

### Desenvolvimento do Instrumento de Auditoria

O instrumento de auditoria do ambiente alimentar baseado na classificação NOVA e nomeado AUDITNOVA foi planejado em etapas que envolveram reuniões sistemáticas do grupo de pesquisa; análises detalhadas dos instrumentos de auditoria brasileiros e internacionais e análise detalhada da classificação de alimentos NOVA proposta por Monteiro et al.^17^ . O processamento de alimentos identificado pela NOVA envolve processos físicos, biológicos e químicos empregados depois que os alimentos são separados da natureza e antes de serem consumidos ou preparados como pratos e refeições. A NOVA classifica todos os alimentos em quatro grandes grupos:

- grupo 1: alimentos *in natura* ou minimamente processados. *In natura* são definidos como partes comestíveis de plantas ou animais após deixarem a natureza e minimamente processados são alimentos *in natura* alterados por processos que incluem remoção de partes não comestíveis ou não desejadas e secagem, esmagamento, moagem, fracionamento, filtragem, torrefação, ebulição, fermentação não alcoólica, pasteurização, refrigeração, congelamento e embalagem a vácuo;
- grupo 2: ingredientes culinários. Definidos como substâncias derivadas de alimentos do grupo 1 ou da natureza por processos que incluem pressionar, refinar, moer e secar, com o objetivo de fazer produtos duráveis utilizados para cozinhar e preparar alimentos em casa ou em restaurantes;
- grupo 3: alimentos processados. São feitos essencialmente adicionando sal, óleo, açúcar ou outras substâncias do grupo 2 ao grupo 1;
- grupo 4: alimentos ultraprocessados. Definidos como formulações feitas principalmente ou inteiramente a partir de substâncias derivadas de alimentos e aditivos, com pouco ou nenhum alimento do grupo 1^17^ .

Os 66 alimentos selecionados para fazer parte do AUDITNOVA foram aqueles que apresentaram maior frequência de aquisição pela população brasileira segundo dados da Pesquisa de Orçamentos Familiares (POF) 2008-2009^21^ . A primeira versão do instrumento foi composta por dois blocos, um sobre disponibilidade, preços, variedade e qualidade dos alimentos e outro sobre publicidade e propaganda.

A primeira versão do instrumento passou pelo processo de validação de conteúdo por um painel de juízes com a participação de nove especialistas distribuídos entre as seguintes áreas de atuação: pesquisa em ambiente alimentar, publicidade e propaganda de alimentos e defesa do consumidor. O referencial teórico adotado durante o painel de juízes foi o GAPB, especialmente o Capítulo 2 (“A escolha dos alimentos”) e o Capítulo 5 (“A compreensão e superação dos obstáculos”). Cada item do AUDITNOVA foi analisado pelos juízes, que atribuíram pontuações baseadas na escala de Likert de 4 pontos (1: não está de acordo, 2: maiores revisões são necessárias, 3: pequenas revisões são necessárias e 4: está de acordo) para os atributos clareza, relevância, pertinência e representatividade. Além disso, os juízes dispunham de um campo para escrever sugestões caso a pontuação fosse igual ou inferior a 3. Foi calculado o índice de validade de conteúdo (claro, relevante, pertinente e representativo) para cada item e para cada bloco do instrumento. O IVC foi considerado satisfatório quando alcançou concordância de 0,80 entre os juízes. Os itens que não atingiram tal porcentagem foram descartados da versão final do instrumento^22^ .

A versão final do AUDITNOVA, após análise pelo painel de juízes, contém 14 blocos de questões divididos da seguinte maneira: bloco 1 – informações gerais (identificação do município; identificação do avaliador; identificação do estabelecimento comercial (número sequencial atribuído pelo pesquisador); nome e endereço do estabelecimento comercial; data, hora de início e hora final da coleta); bloco 2 – tipo de comércio e produtos comercializados (tipo de estabelecimento comercial, grupos alimentares comercializados segundo a NOVA, grupos alimentares comercializados prioritariamente segundo a NOVA); bloco 3 – entrada do estabelecimento (seção de frutas e hortaliças na entrada da loja, disponibilidade de alimentos nos caixas, disponibilidade de propagandas nos caixas); bloco 4 – setor de hortifrútis (disponibilidade, unidade de medida, preço atual em reais/quilo e tipo de preço – normal ou promocional); bloco 5 – setor de carnes, frangos e peixes (disponibilidade, preço mais caro em reais/quilo, preço mais barato em reais/quilo e tipo de preço); bloco 6 – setor de laticínios (disponibilidade, quantidade de marcas disponíveis, preço mais caro, preço mais barato e tipo de preço); bloco 7 – setor de mercearia (disponibilidade, quantidade de marcas disponíveis, preço mais caro, preço mais barato e tipo de preço); bloco 8 – setor de latarias e conservas (disponibilidade, quantidade de marcas disponíveis, preço mais caro, preço mais barato e tipo de preço); bloco 9 – setor de panificados e matinais (disponibilidade, quantidade de marcas disponíveis, preço mais caro, preço mais barato e tipo de preço); bloco 10 – setor de congelados (disponibilidade, quantidade de marcas disponíveis, preço mais caro, preço mais barato e tipo de preço); bloco 11 – setor de bebidas (disponibilidade, quantidade de marcas disponíveis, preço mais caro, preço mais barato e tipo de preço); bloco 12 – setor de chocolates, bolachas e salgadinhos de pacote (disponibilidade, quantidade de marcas disponíveis, preço mais caro, preço mais barato e tipode preço); e, por fim, os blocos 13 e 14, sobre propagandas dentro e fora do estabelecimento, respectivamente (Arquivo Suplementar).

### Reprodutibilidade e Confiabilidade do Instrumento de Auditoria

O estudo de avaliação da confiabilidade do AUDITNOVA foi realizado em uma amostra de conveniência da região metropolitana da cidade de São Paulo (SP), de fácil acesso por transporte público a partir da Faculdade de Saúde Pública. A seleção dos bairros foi projetada para maximizar a capacidade de contrastar supermercados em bairros com diferentes níveis de renda. Para garantir diferenças socioeconômicas entre eles, foram escolhidos locais com diferentes índices de desenvolvimento humano (IDH): Pinheiros (IDH = 0,98), Higienópolis (IDH = 0,93), Belém (IDH = 0,91) e Sacomã (IDH = 0,84). Em cada bairro foram selecionados 20 estabelecimentos comerciais dos tipos supermercado, hipermercado e mercado, totalizando uma amostra de 80 estabelecimentos. Apenas sete não aceitaram participar da pesquisa. Os supermercados, hipermercados e mercados apresentam grande variedade de produtos alimentícios disponíveis ao consumidor^23 , 24^ , sendo equipamentos importantes para medir a confiabilidade e reprodutibilidade de um instrumento de auditoria do ambiente alimentar, visto que possibilitam ao pesquisador a aplicação da ferramenta de forma completa.

Para avaliar a confiabilidade interavaliadores, dois pesquisadores treinados visitaram de forma independente os 73 estabelecimentos comerciais da região escolhida para o estudo. A confiabilidade interavaliadores é utilizada para avaliar a consistência de uma medida realizada por avaliadores distintos. Para avaliar a confiabilidade teste-reteste, um total de 41 estabelecimentos foram revisitados pelos mesmos pesquisadores 32 dias após as observações iniciais. A confiabilidade teste-reteste é usada para avaliar a consistência de uma medida entre dois momentos distintos.

Para as variáveis categóricas, a confiabilidade interavaliadores e teste-reteste foi aferida por percentual de concordância e coeficientes de Kappa. Os valores de Kappa foram quantificados usando a seguinte escala: 0,01 a 0,20, ligeira concordância; 0,21 a 0,40, concordância justa; 0,41 a 0,60, concordância moderada; 0,61 a 0,80, concordância substancial; 0,81 a 0,99, alta concordância^25^ . Os valores de concordância negativos foram interpretados como sinal de que os avaliadores concordaram menos em um item do que o esperado devido ao acaso – por exemplo, houve um desacordo sistemático entre os observadores devido à diversidade de itens de alimentos disponíveis nos supermercados. Para avaliar a confiabilidade da disponibilidade de alimentos segundo a NOVA, os 66 itens alimentares presentes no instrumento foram agrupados da seguinte forma: grupo 1 – somatória de todos os alimentos *in natura* /minimamente processados; grupo 2 – somatória de todos os ingredientes culinários; grupo 3 – somatória de todos os alimentos processados e grupo 4 – somatória de todos os alimentos ultraprocessados.

Para as variáveis quantitativas, como preço e quantidade de marcas disponíveis, primeiro foi realizada uma análise exploratória com o uso de *scatter-plots* ilustrando o ajuste linear e o ajuste quadrático. *Scatter-plots* são estratégias que permitem identificar padrões na distribuição dos dados e possíveis erros sistemáticos e aleatórios dependendo de como os pontos estão distribuídos ao longo da reta^26^ . Subsequentemente, foi calculado o coeficiente de correlação de Pearson (r), que mede o grau da correlação linear entre duas variáveis quantitativas. É um índice adimensional com valores situados ente -1.0 e 1.0, que reflete a intensidade de uma relação linear entre dois conjuntos de dados. O coeficiente de correlação de Pearson foi calculado entre os pares de variáveis coletadas pelo pesquisador 1 e pelo pesquisador 2 e entre as variáveis coletadas na primeira e na segunda visita. Foram calculados a média de preço e de marcas para cada um dos quatro grupos de alimentos analisados. As estatísticas foram realizadas no pacote estatístico Stata 14.

## RESULTADOS

Na etapa de validação de conteúdo realizada por nove juízes, os especialistas forneceram informações necessárias para revisar o instrumento de auditoria e melhorar seu conteúdo. O índice de validade de conteúdo, que representa a média da pontuação para os atributos clareza, relevância, pertinência e representatividade, foi de 0,91 para o instrumento todo. Apesar de a maioria dos itens terem IVC maior que 0,80, as sugestões fornecidas pelos especialistas foram incorporadas ao instrumento por serem totalmente pertinentes segundo avaliação do pesquisador ( Tabela 1 ).

**Tabela 1:** Resultados da validação de conteúdo do AUDITNOVA: mudanças realizadas e motivos para as mudanças.

Item	IVC médio*	Mudança	Motivos para a mudança
Bloco 1 – disponibilidade, preço, variedade e qualidade dos alimentos			

Item 1. Dos alimentos *in natura* /minimamente processados, quais o local comercializa?	0,97	Inclusão de novos alimentos *in natura* e divisão dos itens alimentares conforme seções presentes nos supermercados	Auditar alimentos *in natura* /minimamente processados frequentemente consumidos pela população brasileira e facilitar a coleta de dados
Item 2. Seção de FLV localizada próximo à entrada da loja?	0,97	Nenhuma	-
Item 3. Alimentos *in natura* /minimamente processados: disponibilidade e preço	0,89	Inclusão de opções para tipo de preço (normal ou promocional) e unidade de medida para FLV (kg, dúzia, unidade); remoção do item qualidade do alimento	Variação de unidades de medida dificultando a coleta de preços; baixa capacidade de distinção de qualidade do alimento
Item 4. Dos ingredientes culinários, quais o local comercializa?	0,89	Inclusão de novos itens; distribuição dos itens por seção do supermercado	Auditar ingredientes culinários frequentemente consumidos pela população brasileira e facilitar a coleta de dados
Item 5. Ingredientes culinários: disponibilidade e preço	0,97	Inclusão do tipo de preço (normal ou promocional), quantidade de marcas, preço mais caro e mais barato; padronização dos itens	Auditar mais detalhes dos ingredientes culinários
Item 6. Dos alimentos processados, quais o local comercializa?	0,97	Inclusão de novos itens; distribuição dos itens por seção do supermercado	Auditar alimentos processados frequentemente consumidos pela população brasileira e facilitar a coleta de dados
Item 7. Alimentos processados: disponibilidade e preço	0,92	Inclusão do tipo de preço (normal ou promocional), quantidade de marcas, preço mais caro e mais barato; padronização dos itens	Auditar mais detalhes dos alimentos processados
Item 8. Dos alimentos ultraprocessados, quais o local comercializa?	0,94	Inclusão de novos itens; distribuição dos itens por seção do supermercado	Auditar alimentos ultraprocessados frequentemente consumidos pela população brasileira e facilitar a coleta de dados
Item 9. Alimentos ultraprocessados: disponibilidade e preço	0,90	Inclusão do tipo de preço (normal ou promocional), quantidade de marcas, preço mais caro e mais barato; padronização dos itens	Auditar mais detalhes dos alimentos ultraprocessados

Bloco 2 – publicidade e propagandas			

Item 1. Propaganda visual que incentiva a compra de FLV	0,88	Excluído e transformado em novos itens	Os itens novos incorporados deram conta de captar a variabilidade de propagandas
Item 2. Tipo de propaganda verificada na seção de FLV	0,89	Cada tipo virou um novo item no questionário	Os itens novos incorporados deram conta de captar a variabilidade de propagandas
Item 3. Apelo e motivo da propaganda na seção de FLV	0,97	Cada apelo e motivo virou um novo item no questionário	Os itens novos incorporados deram conta de captar a variabilidade de propagandas
Item 4. Propaganda visual que incentiva a compra de bebidas açucaradas como sucos adicionados de açúcar, néctar e refrigerantes	0,94	Excluído e transformado em novos itens para avaliar propaganda de ultraprocessados em geral	Os itens novos incorporados deram conta de captar a variabilidade de propagandas
Item 5. Tipos de propagandas de bebidas açucaradas	0,92	Excluído e transformado em um item para avaliar propaganda de ultraprocessados em geral	Os itens novos incorporados deram conta de captar a variabilidade de propagandas
Item 6. Apelo e motivo da propaganda na seção de bebidas açucaradas	0,89	Os apelos e motivos mais relevantes viraram novos itens no questionário	Os itens novos incorporados deram conta de captar a variabilidade de propagandas
Item 7. Propaganda visual que incentiva a compra de biscoitos, bolachas e salgadinhos de pacote nas seções onde esses alimentos podem ser encontrados	0,92	Excluído e transformado em um item para avaliar propaganda de ultraprocessados em geral	Os itens novos incorporados deram conta de captar a variabilidade de propagandas
Item 8. Tipo de propagandas de biscoitos/bolachas/salgadinhos	0,86	Excluído e transformado em um item para avaliar propaganda de ultraprocessados em geral	Os itens novos incorporados deram conta de captar a variabilidade de propagandas
Item 9. Apelo e motivo da propaganda na seção de biscoitos/bolachas/salgadinhos	0,86	Os apelos e motivos mais relevantes viraram novos itens no questionário	Os itens novos incorporados deram conta de captar a variabilidade de propagandas
Item 10. Há mensagens de promoção da saúde ou publicidade relacionadas com a temática alimentação e saúde no estabelecimento?	0,83	Sem alteração, porém aplicada para alimentos *in natura* /minimamente processados e ultraprocessados	Essa temática se aplica a diversos tipos de alimentos, saudáveis e não saudáveis
Item 11. Escreva abaixo as mensagens	0,81	Excluído	Os itens novos incorporados deram conta de captar a variabilidade de propagandas
Item 12. Os caixas possuem revistas/cartazes/folders com notícias sobre dietas da moda, superalimentos, diet versus light e/ou outras notícias sobre alimentação e saúde?	0,97	Nenhuma	-
Item 13. Os caixas comercializam chocolates, guloseimas, refrigerantes, energéticos ou outros tipos de produtos ultraprocessados?	0,92	Cada alimento foi detalhado em um novo item	Caracterizar com mais detalhes a área de *check-out*
Item 14. Os caixas apresentam propaganda de alimentos ultraprocessados?	0,94	Nenhuma	-

IVC total do instrumento	0,91	-	-

FLV: frutas, legumes e verduras; *check-out* : áreas onde se localizam os caixas dos estabelecimentos comerciais

* O IVC (índice de validade de conteúdo) representa a média da pontuação atribuída pelos nove juízes especialistas para os atributos clareza, relevância, pertinência e representatividade.

Durante o processo de auditoria, os dois pesquisadores treinados realizaram as primeiras visitas aos 73 estabelecimentos em uma média de 41 dias (desvio padrão = 11,8 dias). O tempo médio de aplicação do AUDITNOVA foi de 90 minutos (desvio padrão = 7,0 minutos). A segunda visita foi realizada pelos pesquisadores entre 32 e 47 dias após a primeira coleta, com uma média de 39,5 dias (desvio padrão = 4,8 dias).

A Tabela 2 mostra os resultados da confiabilidade interavaliadores e teste-reteste para os 66 alimentos auditados e também para os quatro grupos de alimentos segundo a NOVA. A maioria dos alimentos apresentou valores de Kappa maiores que 0,80 (concordância substancial) tanto para interavaliadores quanto para teste-reteste. Analisando os grupos da NOVA, verificou-se que os alimentos *in natura* /minimamente processados apresentaram valores de Kappa moderados (0,41–0,60) tanto para interavaliadores quanto para teste-reteste. Os outros três grupos de alimentos (ingredientes culinários, alimentos processados e alimentos ultraprocessados) apresentaram valores de Kappa acima de 0,70 para a confiabilidade interavaliadores e variando de 0,57 a 0,64 na confiabilidade teste-reteste, o que indica concordância moderada entre as visitas.

**Tabela 2:** Confiabilidade do instrumento AUDITNOVA segundo indicadores de disponibilidade de alimentos.

Tipos de alimentos disponíveis	Confiabilidade interavaliadores			Confiabilidade teste-reteste		
	
	n	% de concordância	Kappa	n	% de concordância	Kappa
Alimentos *in natura* /minimamente processados	72	59,7	0,55	41	55,8	0,52
Laranja	73	100,0	1,00	41	92,7	0,88
Banana	73	94,5	0,72	41	82,9	0,72
Mamão-formosa	72	100,0	1,00	41	78,5	0,61
Maçã-fuji	73	93,1	0,70	41	90,2	0,79
Melancia	72	97,2	0,93	41	85,4	0,74
Outras frutas	73	98,6	0,85	41	97,6	0,94
Tomate-débora	73	93,1	0,83	41	90,2	0,84
Cebola	73	98,6	0,85	41	92,7	0,84
Alface	73	100,0	1,00	41	87,8	0,78
Cenoura	73	98,6	0,90	41	78,0	0,64
Abobrinha	73	91,8	0,75	41	87,8	0,79
Chuchu	73	98,6	0,91	41	92,7	0,86
Cheiro-verde	73	95,9	0,88	41	82,9	0,71
Outras hortaliças	73	97,3	0,78	41	92,7	0,86
Batata-inglesa	73	98,6	0,90	41	85,4	0,72
Mandioca	73	98,6	0,97	41	65,8	0,44
Outras raízes e tubérculos	73	94,5	0,64	41	95,1	0,90
Milho verde	73	94,5	0,87	41	73,2	0,57
Ovo	73	94,5	0,84	41	75,6	0,6
Outros ovos	73	97,3	0,90	41	85,4	0,74
Carne de primeira	73	100,0	1,00	41	87,8	0,81
Carne de segunda	73	98,6	0,97	41	87,8	0,81
Frango	73	98,6	0,96	41	80,5	0,68
Peito de frango	73	98,6	0,85	41	95,1	0,90
Peixe	73	98,6	0,93	41	100,0	1,00
Leite de vaca	73	100,0	1,00	41	97,6	0,95
Feijão-carioca	73	100,0	1,00	41	95,1	0,91
Feijão-preto	73	100,0	1,00	41	90,2	0,82
Arroz branco	73	100,0	1,00	41	97,6	0,95
Farinha de trigo	73	100,0	1,00	41	100,0	1,00
Farinha de mandioca	73	98,6	0,79	41	87,8	0,73
Macarrão espaguete	73	98,6	0,93	41	100,0	1,00
Amendoim cru	73	90,4	0,70	41	75,6	0,56
Água 500 mL	73	100,0	^a^	41	97,6	0,95
Água galão 5 litros	73	100,0	1,00	41	73,2	0,55
Ingredientes culinários	73	93,1	0,87	41	70,7	0,64
Manteiga	73	100,0	1,00	41	92,7	0,86
Óleo de soja	73	100,0	^a^	41	95,1	0,89
Azeite de oliva	73	100,0	1,00	41	97,6	0,93
Sal refinado	73	98,6	0,79	41	100,0	1,00
Açúcar cristal	73	94,5	0,88	41	80,5	0,68
Açúcar refinado	73	100,0	^a^	41	100,0	1,00
Alimentos processados	73	94,5	0,89	41	68,3	0,61
Toucinho	73	98,6	0,91	41	92,7	0,86
Carne-seca	73	97,3	0,93	41	80,5	0,70
Queijo	73	100,0	^a^	41	97,6	0,94
Milho em conserva	73	100,0	1,00	41	97,6	0,95
Extrato de tomate	73	100,0	1,00	41	100,0	1,00
Sardinha em conserva	73	98,6	0,79	41	97,6	0,95
Pão francês	73	100,0	1,00	41	82,9	0,68
Alimentos ultraprocessados	73	82,2	0,76	41	61,0	0,57
Salsicha	73	100,0	1,00	41	95,1	0,91
Linguiça	73	93,1	0,80	41	90,2	0,82
Bebida láctea	73	98,6	0,95	41	92,7	0,86
Macarrão instantâneo	73	98,6	0,88	41	90,2	0,80
Tempero pronto	73	100,0	1,00	41	100,0	1,00
Pão de forma branco	73	97,3	0,78	41	90,2	0,79
Cereais matinais	73	98,6	0,88	41	97,6	0,94
Pizza pronta	73	100,0	1,00	41	95,1	0,90
Sorvete	73	98,6	0,94	41	85,4	0,73
Refrigerante regular lata^a^	73	100,0	1,00	41	100,0	1,00
Refrigerante regular 2 litros^b^	73	100,0	1,00	41	100,0	1,00
Refrigerante zero/light/diet	73	100,0	1,00	41	97,6	0,94
Néctar	73	100,0	1,00	41	100,0	1,00
Refresco	73	100,0	1,00	41	100,0	1,00
Salgadinho	73	100,0	1,00	41	100,0	1,00
Biscoito recheado	73	100,0	^a^	41	100,0	^a^
Chocolate	73	95,9	0,80	41	92,7	0,85
Balas	73	97,3	0,94	41	97,5	0,96

^a^As estatísticas não puderam ser calculadas porque a tabulação cruzada tinha dois ou menos níveis.

^b^Refrigerante com açúcar adicionado.

A Tabela 3 mostra os resultados da confiabilidade interavaliadores e teste-reteste para os indicadores de alimentos comercializados e prioritariamente comercializados segundo a NOVA, seção de frutas e hortaliças na entrada do estabelecimento comercial e tipo de preço (normal ou promocional) para os alimentos agrupados segundo a NOVA. Apesar das altas concordâncias observadas para todos os indicadores, valores de Kappa razoáveis (0,21–0,40) e moderados (0,41–0,60) foram observados tanto na confiabilidade interavaliadores quanto teste-reteste, mostrando a capacidade baixa de concordância de alguns deles, como comercialização prioritária de alimentos processados e tipo de preço de alimentos ultraprocessados.

**Tabela 3:** Confiabilidade do instrumento AUDITNOVA segundo indicadores de disponibilidade de alimentos e tipos de preço segundo a NOVA.

Indicadores da disponibilidade de grupos alimentares e tipo de preço segundo a NOVA	Confiabilidade interavaliadores			Confiabilidade teste-reteste		

	n	% de concordância	Kappa	n	% de concordância	Kappa
F&H na entrada da loja	73	95,9	0,92	41	97,6	0,95
Grupos de alimentos comercializados						
*In natura* /minimamente processados	73	100,0	*	41	100,0	*
Ingredientes culinários	73	100,0	*	41	100,0	*
Alimentos @processados	73	100,0	*	41	100,0	*
Alimentos ultraprocessados	73	100,0	*	41	100,0	*
Grupos de alimentos prioritariamente comercializados						
*In natura* /minimamente processados	73	75,3	0,69	41	90,2	0,78
Ingredientes culinários	73	97,3	0,75	41	95,1	0,88
Alimentos processados	73	87,7	0,36	41	95,1	0,90
Alimentos ultraprocessados	73	91,8	0,78	41	100,0	1,00
Tipo de preço (normal ou promocional)						
*In natura* /minimamente processados	73	93,0	0,90	41	93,6	0,89
Ingredientes culinários	73	81,1	0,77	41	82,1	0,78
Alimentos processados	73	71,4	0,61	41	70,4	0,58
Alimentos ultraprocessados	73	61,7	0,56	41	52,6	0,48

F&H: frutas e hortaliças

* As estatísticas não puderam ser calculadas porque a tabulação cruzada tinha dois ou menos níveis.

Na Tabela 4 são apresentados os resultados da confiabilidade interavaliadores e teste-reteste para as variáveis de publicidade e propaganda presentes no AUDITNOVA. Foi possível observar que a confiabilidade interavaliadores obteve uma maior quantidade de coeficientes de Kappa acima de 0,80 do que a teste-reteste. Para a confiabilidade interavaliadores, os valores de Kappa variaram de 0,57 a 1,00, e para a teste-reteste, de 0,18 a 0,90, mostrando a maior discordância nesta.

**Tabela 4:** Confiabilidade do instrumento AUDITNOVA segundo indicadores de propaganda e estratégias publicitárias de alimentos segundo a classificação NOVA.

Tipos de propagandas e estratégias publicitárias	Confiabilidade interavaliadores			Confiabilidade teste-reteste		
	
	n	% de concordância	Kappa	n	% de concordância	Kappa
Propagandas na área de *check-out*						
Propagandas de diversos tipos com *in natura* /minimamente processados	73	89,0	0,57	41	90,2	0,77
Propaganda de diversos tipos com AUP	73	93,1	0,81	41	92,7	0,83
Tabloide	73	94,5	0,90	41	94,5	0,90
Folder ou folheto	73	98,6	0,88	41	95,1	0,79
Abordagens no espaço físico						
Bandeirolas com *in natura*	73	87,7	0,65	41	87,8	0,72
Banner/cartazes com *in natura*	72	84,7	0,60	41	82,5	0,52
Displays com *in natura*	73	89,0	0,65	41	87,8	0,46
Tabloides com *in natura*	73	87,7	0,75	41	65,8	0,39
Folder/folheto com receitas *in natura*	73	91,8	0,65	41	90,2	0,55
Displays com AUP	73	100,0	1,00	41	95,2	*
Ilhas promocionais com AUP	73	86,3	0,67	41	68,3	0,37
Ponta de gôndola com AUP	73	82,2	0,66	41	72,5	0,48
Balcão de degustação com AUP	73	97,3	0,74	41	95,1	*
Tipos de mensagens e apelos ao consumidor						
Propriedade funcional com *in natura*	73	98,6	0,95	41	87,8	0,72
Atividade física e *in natura*	73	98,6	0,66	41	95,1	0,62
Bem-estar, bom-humor, autoestima com *in natura* /minimamente processados	73	91,8	0,62	41	92,7	0,70
Alegação de saúde com *in natura*	72	95,8	0,84	41	82,9	0,72
Apelo à praticidade com *in natura* /minimamente processados	73	94,5	0,68	41	92,7	0,67
Destaque de sabor, odor, cor ou textura com *in natura* /minimamente processados	72	94,4	0,84	41	87,8	0,48
Alegação de saúde e bem-estar com AUP	73	86,3	0,72	41	78,0	0,58
Apelo à praticidade com AUP	73	84,9	0,62	41	56,1	0,23
Propriedade funcional com AUP	73	84,9	0,68	41	72,5	0,41
Destaque de sabor, odor, cor ou textura com AUP	73	90,4	0,80	41	65,8	0,43
Propagandas relacionadas a degustações, brindes, promoções e lançamentos						
Leve 3, pague 2 com *in natura*	73	98,6	*	41	97,6	*
Lançamentos de produtos *in natura* /minimamente processados	72	98,6	*	41	*	*
Brindes ou venda casada com *in natura* /minimamente processados	73	95,9	0,64	41	*	*
Amostra grátis de AUP	73	98,6	*	41	95,6	*
Leve 3, pague 2 com AUP	73	87,7	0,71	41	78,0	0,46
Lançamentos de AUP	73	78,1	0,62	41	53,6	0,18
Brindes ou venda casada com AUP	73	79,4	0,61	41	73,2	0,46
Propaganda geral com ingredientes culinários	73	86,3	0,70	41	61,0	0,18
Propaganda geral com processados	73	80,8	0,65	41	61	0,20
Propagandas no exterior do estabelecimento						
Propagandas em geral com *in natura* /minimamente processados	73	90,4	0,70	41	78,0	0,61
Propaganda em geral de ingredientes culinários	73	94,5	0,77	41	80,5	0,66
Propaganda em geral alimentos processados	73	94,5	0,82	41	75,6	0,58
Propaganda fora do estabelecimento com AUP	73	93,1	0,85	41	78,5	0,60

*check-out* : áreas onde se localizam os caixas dos estabelecimentos comerciais; AUP: alimentos ultraprocessados

* As estatísticas não puderam ser calculadas porque a tabulação cruzada tinha dois ou menos níveis.

Os *scatter-plots* ilustrando a relação entre as variáveis de preço de alimentos segundo a NOVA coletadas pelos pesquisadores 1 e 2 e coletadas na primeira e segunda visita são apresentadas na Figura 1 . As inclinações das retas mostram uma correlação positiva em todos os casos.

**Figura 1:** Relações entre as variáveis de preços dos alimentos segundo a NOVA coletadas pelos pesquisadores 1 e 2 coletadas na primeira e na segunda visita.

Pesq.: pesquisador; in nat/min.processados: *in natura* e minimamente processados

A Figura 2 mostra os *scatter-plots* ilustrando a relação entre as variáveis de quantidade de marcas de alimentos segundo a NOVA coletadas pelos pesquisadores 1 e 2 e coletadas na primeira e segunda visita. As inclinações das retas mostram uma correlação positiva em todos os casos.

**Figura 2:** Relações entre as variáveis de quantidade de marcas dos alimentos segundo a NOVA coletadas pelos pesquisadores 1 e 2 coletadas na primeira e na segunda visita.

Pesq: pesquisador; min. processados: minimamente processados

A Tabela 5 mostra os valores médios de preço e quantidade de marcas encontrados pelos pesquisadores 1 e 2 em cada visita, assim como os valores da correlação de Pearson (r) dos pares de variáveis analisadas. Embora todos os valores de correlação tenham sido positivos, a quantidade de marcas de alimentos minimamente processados apresentou os valores de r mais baixos tanto entre os avaliadores quanto entre as visitas realizadas.

**Tabela 5:** Média e desvio-padrão (DP) do preço e quantidade de marcas coletados pelos pesquisadores 1 e 2 e nas visitas 1 e 2 e valores do coeficiente de correlação de Pearson (r) entre os pares de variáveis.

Variáveis quantitativas	Interavaliadores			Teste-reteste		
	
	Média (DP) pesquisador 1	Média (DP) pesquisador 2	r	Média (DP) visita 1	Média (DP) visita 2	r
Preço *in natura* /minimamente processados	5,34 (1,51)	5,28 (1,42)	0,98	5,51 (1,26)	5,35 (1,47)	0,82
Preço ingredientes	7,17 (2,68)	7,15 (2,61)	0,99	7,12 (2,66)	7,11 (2,86)	0,90
Preço processados	16,14 (8,41)	16,34 (8,59)	0,97	15,09 (4,23)	15,04 (4,28)	0,65
Preço ultraprocessados	6,85 (1,53)	6,67 (1,59)	0,95	6,93 (1,60)	6,63 (1,36)	0,84
Marcas minimamente processados	3,45 (1,19)	3,51 (1,62)	0,75	3,91 (1,73)	3,46 (1,10)	0,47
Marcas ingredientes	3,88 (2,05)	3,85 (2,01)	0,98	3,91 (1,89)	4,01 (2,09)	0,93
Marcas processados	3,09 (1,18)	3,10 (1,23)	0,96	3,35 (1,15)	3,13 (1,20)	0,89
Marcas ultraprocessados	4,19 (1,35)	4,00 (1,21)	0,97	4,32 (1,06)	4,24 (1,11)	0,92

## DISCUSSÃO

O instrumento de auditoria do ambiente alimentar desenvolvido neste estudo, AUDITNOVA, teve alta confiabilidade interavaliadores e teste-reteste, o que garante que ele seja um instrumento confiável para pesquisas que objetivam trabalhar com indicadores do ambiente alimentar baseados na classificação NOVA de alimentos proposta por Monteiro et al.^17^ . Os alimentos indicadores dos quatro grupos propostos na NOVA foram cuidadosamente selecionados por serem frequentemente adquiridos pelos brasileiros segundo pesquisas nacionais e também recomendados pelo GAPB. Esses alimentos incluídos no AUDITNOVA poderão fornecer uma avaliação dos estabelecimentos varejistas no que diz respeito à disponibilidade de alimentos saudáveis e não saudáveis segundo as diretrizes nacionais^12^ . Além disso, as informações sobre preço, quantidade de marcas e publicidade possibilitarão avaliar o ambiente alimentar do consumidor com detalhes, observando as barreiras e facilidades que os consumidores enfrentam ao realizar suas escolhas alimentares^1^ . A grande maioria dos indicadores presentes neste instrumento é apropriada para o planejamento de programas políticos com o objetivo de modificar o ambiente, avaliar as necessidades de intervenção e as necessidades da população perante a disponibilidade de alimentos e servir como indicadores de avaliação, vigilância e *advocacy* para outras ações pautadas no ambiente alimentar do consumidor^20^ .

A alta confiabilidade entre avaliadores indica que as definições e instruções no manual de medição e os métodos de treinamento foram suficientes para preparar os observadores para coletar dados de alta qualidade. A alta confiabilidade teste-reteste na maioria dos indicadores analisados sugere que ocorrem apenas pequenas mudanças na disponibilidade, preço, quantidade de marcas e estratégias publicitárias dos alimentos ao longo do período de coleta de dados. Assim, as medidas coletadas com o AUDITNOVA geraram uma estimativa estável sobre o ambiente alimentar do consumidor. No entanto, a disponibilidade e o preço dos alimentos *in natura* /minimamente processados geralmente mudam ao longo das estações; portanto, sempre que o instrumento for reaplicado, deverá ser levada em conta a repetição das observações para avaliar ou controlar os efeitos sazonais^14^ .

No Brasil, estudos sobre o ambiente alimentar são recentes, e os instrumentos pioneiros para auditoria do ambiente alimentar comunitário e ambiente alimentar do consumidor foram desenvolvidos e validados por Martins et al.^15^ e Duran et al.^16^ . O instrumento de auditoria validado por Martins et al.^15^ é uma adaptação do instrumento de auditoria desenvolvido por Glanz et al.^14^ para mensurar o ambiente alimentar do consumidor, especificamente os comércios varejistas de alimentos, e avalia aspectos como disponibilidade, preço e qualidade dos alimentos com uma lista de alimentos orientada pela pirâmide alimentar e pelo grau de processamento, mas não aborda a versão completa da classificação NOVA. O instrumento de auditoria proposto por Duran et al.^16^ foi desenvolvido para auditar estabelecimentos comerciais varejistas de alimentos e restaurantes e mensura aspectos como disponibilidade, variedade, qualidade, preço e publicidade de indicadores de alimentos saudáveis como frutas e hortaliças e indicadores de alimentos não saudáveis como os alimentos ultraprocessados. As principais diferenças do AUDITNOVA desenvolvido e validado neste estudo em relação aos outros dois instrumentos brasileiros foram a utilização integral da classificação NOVA no processo de seleção dos itens alimentares, a ampliação das mensurações sobre publicidade e estratégias promocionais por grupos alimentares, a disponibilidade de 66 itens alimentares (incluindo ingredientes culinários e alimentos processados), a inclusão de aspectos estratégicos do ambiente alimentar do consumidor (como as áreas dos caixas, pontas de gôndolas e ilhas) e a coleta de informações sobre preços normais ou promocionais, fatores de reconhecida importância como determinantes no processo de aquisição de alimentos pela população^1 , 9 , 14 , 20^ .

Os principais indicadores propostos neste instrumento apresentaram valores de Kappa substanciais e altos. Foi possível observar que o indicador de disponibilidade de alimentos *in natura* /minimamente processados apresentou valores de Kappa moderados, especialmente no teste-reteste, mas, na avaliação dos itens alimentares de forma isolada, os valores de Kappa foram substanciais. No entanto, a sazonalidade e a pouca variedade de alimentos *in natura* /minimamente processados em supermercados e mercados em relação às feiras livres, varejões e sacolões pode ter influenciado a confiabilidade do indicador^24 , 27^ .

O AUDITNOVA possibilita mensurar de forma detalhada as diversas fontes de informações sobre alimentação disponíveis no ambiente alimentar do consumidor, dividindo os tipos de publicidade e propaganda conforme os quatro grupos de alimentos da NOVA classificação. O Guia Alimentar para a População Brasileira reconhece que a publicidade e as informações disponíveis no ambiente alimentar do consumidor podem se tornar um obstáculo para que a população alcance as recomendações alimentares preconizadas^12^ , pois as grandes indústrias de alimentos, especialmente as de alimentos ultraprocessados, utilizam as propagandas para vender mais produtos, não para educar o consumidor^28^ .

A Organização Mundial da Saúde também reconhece que as campanhas massivas de publicidade adotadas pelas indústrias alimentícias, em especial as direcionadas ao público infantil e com diferentes apelos (saúde, boa forma, praticidade, lançamentos, personagens infantis, entre outros) têm um impacto negativo na saúde desses indivíduos. Assim, caberia aos países rever os processos regulatórios a respeito da veiculação dessas propagandas nas embalagens e nas grandes mídias^28^ . Nesse sentido, desenvolver instrumentos de auditoria que possibilitem traçar um panorama destas práticas publicitárias no ambiente alimentar do consumidor e corroborem com o GAPB será imprescindível para o avanço das políticas públicas e regulação. Indicadores de ambiente alimentar que permitam produzir mais evidências sobre sua influência fazem parte da estratégia do enfrentamento da obesidade e DCNT^5^ .

As variáveis de publicidade aferidas pelo AUDITNOVA apresentaram maior confiabilidade interavaliadores do que no teste-reteste, incluindo muitos valores que não puderam ser computados devido à baixa disponibilidade de propagandas nos estabelecimentos. Tal fato, também observado no estudo de Duran et al.^16^ , pode indicar a dificuldade do pesquisador em identificar no comércio varejista as diferentes estratégias publicitárias disponíveis e de saber distinguir, em especial, os tipos de apelos que essas propagandas trazem. As propagandas que apresentaram valores de Kappa inferior a 0,40 no teste-reteste foram: tabloides com propagandas de alimentos *in natura* /minimamente processados, ilhas promocionais com alimentos ultraprocessados, apelo à praticidade com alimentos ultraprocessados, lançamentos de ultraprocessados e propagandas em geral de ingredientes culinários e alimentos processados. Uma hipótese para melhorar a confiabilidade desse indicador seria realizar mais de uma capacitação dos pesquisadores de campo ao longo do processo de auditoria para reafirmar os diferentes tipos de apelo e abordagens das propagandas de alimentos no comércio varejista e/ou ampliar a amostra de estabelecimentos auditados para aumentar a prevalência desses tipos de propaganda. No entanto, tal fato não invalida a utilização do instrumento por pesquisadores da área. Por ser construído em blocos independentes, eles serão livres para selecionar os indicadores que sejam mais adequados aos objetivos de suas pesquisas.

As variáveis preço e quantidade de marcas apresentaram correlações positivas entre as medidas realizadas pelos pesquisadores 1 e 2 e nas visitas 1 e 2. Ambas as variáveis preço e quantidade de marcas influenciam o consumidor no momento da compra de alimentos^29^ . Conseguir mensurar esses aspectos de forma confiável, inclusive ao longo de um determinado intervalo de tempo, é primordial para que o instrumento possa ser utilizado no monitoramento e mapeamento desses indicadores em diversos estabelecimentos comerciais e em diferentes realidades sociais.

Apesar de este estudo não avaliar o ambiente alimentar ao longo do ano, sabe-se que em certos períodos (por exemplo, Natal, Páscoa, dia dos pais e dia das mães), os indicadores de preço, disponibilidade e especialmente publicidade ^a^ podem variar além do esperado devido às campanhas publicitárias e novos produtos disponíveis nessas datas. Portanto, é necessário que o pesquisador avalie a necessidade de aplicação do instrumento nesses períodos.

Entre os pontos fortes do estudo, estão o processo de validação de conteúdo elaborado por um painel de juízes especialistas em ambiente alimentar e publicidade de alimentos e a utilização da classificação de alimentos NOVA como referencial teórico e analítico. Além disso, a utilização de bases de dados nacionais como a POF forneceu subsídios para a seleção de alimentos que fazem parte dos alimentos frequentemente adquiridos pela população brasileira. Outro ponto forte do trabalho é a presença de alimentos em maior variedade em relação aos instrumentos nacionais, possibilitando o agrupamento conforme a NOVA, além da inclusão de informações mais completas sobre publicidade, preços e quantidade de marcas, que poderá fornecer um panorama mais detalhado do ambiente alimentar aos pesquisadores que farão uso do instrumento.

Uma das limitações deste estudo é a amostra de conveniência de uma única cidade brasileira e a baixa variedade de tipos de estabelecimentos comerciais auditados (somente supermercados, hipermercados e mercados). Essa amostra não é representativa do município e do país; entretanto, os bairros apresentam variações socioeconômicas importantes, que podem impactar na disponibilidade de alimentos auditada. Outra limitação é que não avaliamos as diferenças sazonais durante o ano. O instrumento avaliou somente os comércios varejistas utilizados pela população para aquisição de alimentos e não para consumo imediato, como bares e restaurantes. Devido ao grande número de indivíduos que se alimenta fora de casa no Brasil^21^ , se faz necessário o desenvolvimento e validação de instrumentos adequados para auditar esses locais segundo as novas recomendações alimentares nacionais. Neste artigo não foi apresentado um indicador de qualidade de estabelecimentos varejistas de alimentos baseado em possíveis pontuações geradas pelo instrumento, fato reconhecido como importante e que será considerado para futuros estudos.

O instrumento desenvolvido, AUDITNOVA, se mostrou confiável para a realização de auditorias no ambiente alimentar, em especial no ambiente alimentar do consumidor, pois permite traçar um panorama de tipos de equipamentos varejistas no território e analisar de forma ampla os principais determinantes que contribuem para apoiar a população a realizar escolhas alimentares mais saudáveis. O AUDITNOVA é confiável para mensurar aspectos como disponibilidade, preço, quantidade de marcas e publicidade de alimentos. Associações entre o ambiente alimentar, consumo alimentar e obesidade são cada vez mais frequentes, no entanto para chegar nestes resultados são necessários instrumentos confiáveis para coletar dados. O desenvolvimento e validação de um instrumento de auditoria do ambiente alimentar baseado nas recomendações apresentadas no Guia Alimentar para a População Brasileira dialoga com as demais políticas brasileiras e dá suporte ao desenvolvimento de evidências que permitam repensar o papel do ambiente alimentar na disponibilidade, acesso e consequentemente na segurança alimentar e nutricional da população brasileira. O manual de treinamento de coleta de dados desenvolvido nesta pesquisa e o instrumento AUDITNOVA foram publicados e estão disponíveis para download em: http://colecoes.sibi.usp.br/fsp/items/show/3364#?c=0&m=0&s=0&cv=0.

## Footnotes

a Criança e Consumo. Páscoa para quê? Responda prá gente saber! [citado 27 mar 2019]. Disponível em: http://criancaeconsumo.org.br/noticias/pascoaparaque/
